# LC-MS/MS Evaluation of Pyrrolizidine Alkaloids Profile in Relation to Safety of Comfrey Roots and Leaves from Polish Sources

**DOI:** 10.3390/molecules28166171

**Published:** 2023-08-21

**Authors:** Katarzyna Kimel, Sylwia Godlewska, Michał Gleńsk, Katarzyna Gobis, Justyna Ośko, Małgorzata Grembecka, Mirosława Krauze-Baranowska

**Affiliations:** 1 Department of Pharmacognosy with Medicinal Plants Garden, Faculty of Pharmacy, Medical University of Gdańsk, 107 Hallera St., 80-416 Gdansk, Poland; katarzyna.kimel@gumed.edu.pl (K.K.); sylwia.godlewska@gumed.edu.pl (S.G.); 2 Department of Pharmacognosy and Herbal Medicines, Wroclaw Medical University, 211A Borowska St., 50-556 Wrocław, Poland; michal.glensk@umw.edu.pl; 3 Department of Organic Chemistry, Faculty of Pharmacy, Medical University of Gdańsk, 107 Hallera St., 80-416 Gdansk, Poland; katarzyna.gobis@gumed.edu.pl; 4 Department of Bromatology, Faculty of Pharmacy, Medical University of Gdańsk, 107 Hallera St., 80-416 Gdansk, Poland

**Keywords:** pyrrolizidine alkaloids, comfrey, leaves, roots, hepatotoxicity, HPLC-MS/MS, factor analysis

## Abstract

Comfrey (*Symphytum officinale* L.) has a long tradition of use in the treatment of musculoskeletal disorders. However, due to hepatotoxic pyrrolizidine alkaloids (PAs), the EMA restricts the use of comfrey root (CR) to external use only and for short periods of time. Recent studies indicate a low permeability of PAs across the skin, calling into question the safety of topical application of products containing comfrey preparations. The aim of our work was to develop and validate an HPLC method enabling the separation of isomeric PAs from comfrey and, on this basis, to assess the potential toxicity of CR and comfrey leaf (CL) obtained from various Polish sources. The qualitative and quantitative analysis of PAs via HPLC-MS/MS was performed in MRM mode. The results obtained confirmed a lower content of PAs in CL than in CR and showed a wide variation in the composition of PAs in CR, with a much more stable profile of PAs in CL. Factor analysis confirmed that CRs and CLs differ in PA content, which is influenced by the growth conditions and geographical origin. The determined concentrations of PAs prove that in some CRs available on the Polish herbal market, the content of PAs may exceed the daily dose considered safe.

## 1. Introduction

Comfrey (*Symphytum officinale* L., family Boraginaceae) has been used as a medicinal plant for over 2000 years. Indications for the use of comfrey roots include lung diseases, gastric ulcers, bone fractures, joint ailments, muscle pain, and hard-to-heal wounds [1,2]. Modern clinical trials and the results of new research on the biological activity of comfrey root extract confirm its effectiveness in the local treatment of musculoskeletal disorders, such as muscle pain, sprains, and bruises [2,3,4]. Oral use of comfrey products is currently not recommended due to their high content of hepatotoxic pyrrolizidine alkaloids (PAs).

The European Medicines Agency (EMA) permits only the external use of comfrey root (*Symphyti radix*), directly on intact skin in short-term treatments (up to 10 days) and limiting the supply of PA to 0.007 μg/kg (0.35 μg/day) [5]. In the US, the Food and Drug Administration (FDA) in 2001 ordered the withdrawal of all dietary supplements containing comfrey, but it can still be found as an ingredient in some cosmetics [6]. In some countries, such as Germany, Hungary, and Austria, products with comfrey root from which the pyrrolizidine alkaloids have been removed in the process of manufacturing the herbal product are available [3,5]. However, in others, including Poland, the quality and safety of comfrey products is not controlled because they are mainly cosmetics, dietary supplements, or dried herbs–herbal substances. Moreover, despite the regulations allowing comfrey root for medicinal purposes only, products containing comfrey leaf (*Symphyti folium*) are not only widely advertised and recommended on the Internet, but, according to research by Frost et al. [7], also widely prescribed by herbalists—almost half (49.53%) of 179 UK herbalists regularly use comfrey roots and leaves in their practice.

**Figure 1:**
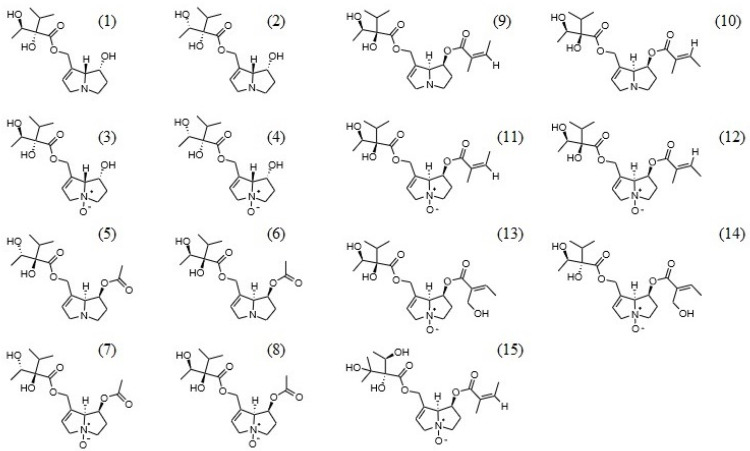
Chemical structures of main pyrrolizidine alkaloids detected in comfrey (*Symphytum officinale*) roots. (**1**)—intermedine, (**2**)—lycopsamine, (**3**)—intermedine *N*-oxide, (**4**)—lycopsamine *N*-oxide, (**5**)—7′-acetylintermedine, (**6**)—7′-acetyllycopsamine, (**7**)—7′-acetylintermedine *N*-oxide, (**8**)—7′-acetyllycopsamine *N*-oxide, (**9**)—symphytine, (**10**)—symlandine, (**11**)—symphytine *N*-oxide, (**12**)—symlandine *N*-oxide, (**13**)—7′-sarracinyl-9-trachelanthylretronecine *N*-oxide, (**14**)—7′-sarracinyl-9-viridiflorylretronecine *N*-oxide, (**15**)—echimidine *N*-oxide.

Although the potential toxicity of comfrey preparations is attributable only to pyrrolizidine alkaloids (Figure 1), data on their composition and content in this plant material are contradictory. According to previous literature, including the 2015 EMA assessment report [5], comfrey root contains the following alkaloids: intermedine/lycopsamine (diastereomers), acetylintermedine/acetyllycopsamine (diastereomers), myoscorpine, lasiocarpine, heliosupine, viridiflorine, echiumine, symlandine and echimidine, and their *N*-oxides. However, more recent studies do not confirm the presence of some of the alkaloids mentioned (myoscorpine, lasiocarpine, heliosupine, echiumine) [8,9,10,11] and indicate their incorrect identification and chemical structure. In addition, the lack of chromatographic separation of diastereomeric pyrrolizidine alkaloids makes their quantification problematic.

The conditions of chromatographic separation given so far in the literature allow us to evaluate their content in pairs as the sum of two stereoisomers [8,11]. This approach can be misleading because it does not take into account differences in their biological activity. Recent studies on the cytotoxicity and hepatotoxicity of the main PAs present in comfrey root—intermedine, lycopsamine, and their *N*-oxides—indicate that lycopsamine and its derivatives are more toxic than the corresponding stereoisomers [12,13]—intermedine and its *N*-oxide.

Recent years have also brought reports suggesting that the current recommendations regarding PAs limits may be overestimated. Although PAs such as intermedine, lycopsamine, and their *N*-oxides have been shown in in vitro models of human and mouse cells to be hepatotoxic [12,13], their permeability through the skin after direct topical application may be very low. It was shown that the penetration of lycopsamine through the human epidermis into the receptor fluid, measured in Franz chambers, was in the range of 0.04–0.22% in the studies of Jedlinszka et al. [14] to 0.6% according to Kuchta et al. [15].

So far, there is little data on the composition and content of PAs in the comfrey leaf, which contains much lower concentrations of pyrrolizidine alkaloids [4]. Betza et al. [16] report that the content of lycopsamine and intermedine in the leaves is 4 times lower than in the roots, while the content of acetyllycopsamine and acetylintermedine is 8- and 11-times lower, respectively. Similar differences were also noted for alkaloid *N*-oxides present in the amount of 0.02–0.18% in the leaves compared to 0.25–0.29% in the roots [17].

In order to ensure the safety of consumers and patients, it is important to develop more effective methods for the separation and quantification of a wide spectrum of pyrrolizidine alkaloids present not only in comfrey but also in other raw plant materials [18,19]. Due to the fact that PAs may be present as impurities in raw medicinal plant materials in very low concentrations, it is difficult to develop an analytical method with appropriate limits of detection (LOD) and quantification (LOQ). The monograph in the European Pharmacopoeia on the LC-MS/MS determination of PAs as impurities in medicinal plant raw materials includes only a few PAs present in comfrey (i.e., lycopsamine, intermedine and their *N*-oxides next to echimidine *N*-oxide) [20].

In the analysis of PAs, GC methods are sometimes used, which must take into account the low volatility of pyrrolizidine alkaloids, which requires reduction of the determined PAs immediately prior to analysis. In addition, there is an increased risk of thermal decomposition in the analyzed PAs, especially *N*-oxides, which are known to be unstable at high temperatures [19]. An additional difficulty is the lack of chromophores in PA structures, which precludes the use of some detection methods [18]. Therefore, due to the high sensitivity, specificity, and selectivity of mass spectrometry hyphenated with HPLC or UHPLC, these systems are currently considered the most suitable for PA analysis [18,19]. The LC-MS/MS method with targeted MRM analysis has proven effective in the identification and quantification of carbazole alkaloids in *Murraya koeniigi* [21].

The aim of our research was to develop an HPLC-MS/MS method enabling effective separation of pyrrolizidine alkaloids, including their stereoisomers, which are present in comfrey root and leaf extracts. The developed method was used to compare the composition of PAs in the roots and leaves of comfrey obtained from various Polish sources. The comparative analysis included identification of alkaloids and semi-quantitative analysis of all identified compounds. In addition, the content of the main pyrrolizidine alkaloids (intermedine, lycopsamine, intermedine *N*-oxide, and lycopsamine *N*-oxide) was quantified by HPLC-MS/MS using the standard addition method. The results obtained confirmed that comfrey leaves contain lower concentrations of PAs and are characterized by a more stable composition of these compounds than comfrey roots.

## 2. Results

### 2.1. Optimization of Separation Conditions

Based on the data from the literature, the separation of PAs was carried out on HPLC column, with the stationary phase being C-18 silica gel, previously used in the analysis of these compounds; however, for the first time a Kinetex column was selected for PA analysis.

In previous publications, separation of pyrrolizidine alkaloids was most often carried out at a column temperature of 40 °C [10,22,23]. However, based on data from Altamirano et al. [11], who observed a significant effect of temperature on the separation of some isomers of pyrrolizidine alkaloids (especially symphytine *N*-oxide), we decided to reduce the temperature to 25 °C during the chromatographic separation. A similar approach was used in the analysis of pyrrolizidine alkaloids in *Symphytum cordatum* [24] and *Echium* spp. [25]. Reducing the oven temperature to 25 °C enabled the separation of intermedine, lycopsamine, and their *N*-oxides and consequently allowed for the quantification of each of these compounds. As with Skoneczny et al. [25], in the profiling of PAs in *Echium* spp., water and a mixture of water:acetonitrile (50:50, *v*/*v*) with the addition of 0.1% formic acid were also used as mobile phase, eliminating the ammonium buffer often used in PA analysis [18]. The gradient elution program was optimized and, compared to the previous HPLC separation of PAs in the roots of *S. officinale* and *Symphytum* × *uplandicum* [11], the concentration of 0.1% formic acid solution in the mixture of water: acetonitrile (50:50, *v*/*v*) was increased from 10% to 70% and the gradient time extended to 55 min, which enabled complete separation of some PA isomers, mainly symphytine and symlandine derivatives.

### 2.2. Identification of Pyrrolizidine Alkaloids

The qualitative analysis of pyrrolizidine alkaloids present in the roots and leaves of comfrey was carried out using the available reference standards—intermedine, lycopsamine, intermedine, and lycopsamine *N*-oxides, and characteristic of their MS^2^ and MS^3^ transitions, described earlier in the literature [8,23,26,27].

Intermedine (**1**) and lycopsamine (**2**) are characterized by slightly different second and third transitions, namely: intermedine—300.1 > 138.05 (*m*/*z*), 300.1 > 156.1 (*m*/*z*), Q(ii)/Q(i) 0.63 and lycopsamine—300.1 > 156.05 (*m*/*z*), 300.1 > 138.1 (*m*/*z*), Q(ii)/Q(i) 0.51, and collision energy of second transition: −20 eV and −19 eV, respectively [8,26]. Similarly, slight differences were observed between intermedine and lycopsamine *N*-oxides, and their retention times indicated that the peak at t_R_ 9.62 min belongs to intermedine *N*-oxide (**3**) and the peak at t_R_ 11.04 min was attributed to lycopsamine *N*-oxide (4) (t_R_ values consistent with reference compounds). The most intensive transition of peaks of both compounds was 316.1 > 172.05 (*m*/*z*), Q(ii)/Q(i) 0.51 [8,26].

Due to the lack of standard compounds among the remaining pyrrolizidine alkaloids, their identification was based on the literature describing the characteristic MS^2^ fragments for each of them [8,23,26,27]. Taking into account the fact that some compounds may not be sufficiently separated, yielding single peaks on the TIC (total ion chromatogram) chromatogram, automatic optimization of ESI ionization conditions was used to determine the correct precursor ion and transition. The applied collision energies were determined on the basis of the highest peak intensities observed for individual transitions. The appropriate collision energies were selected in the range from −15 eV to −45 eV, in steps of 5 eV.

**Figure 2:**
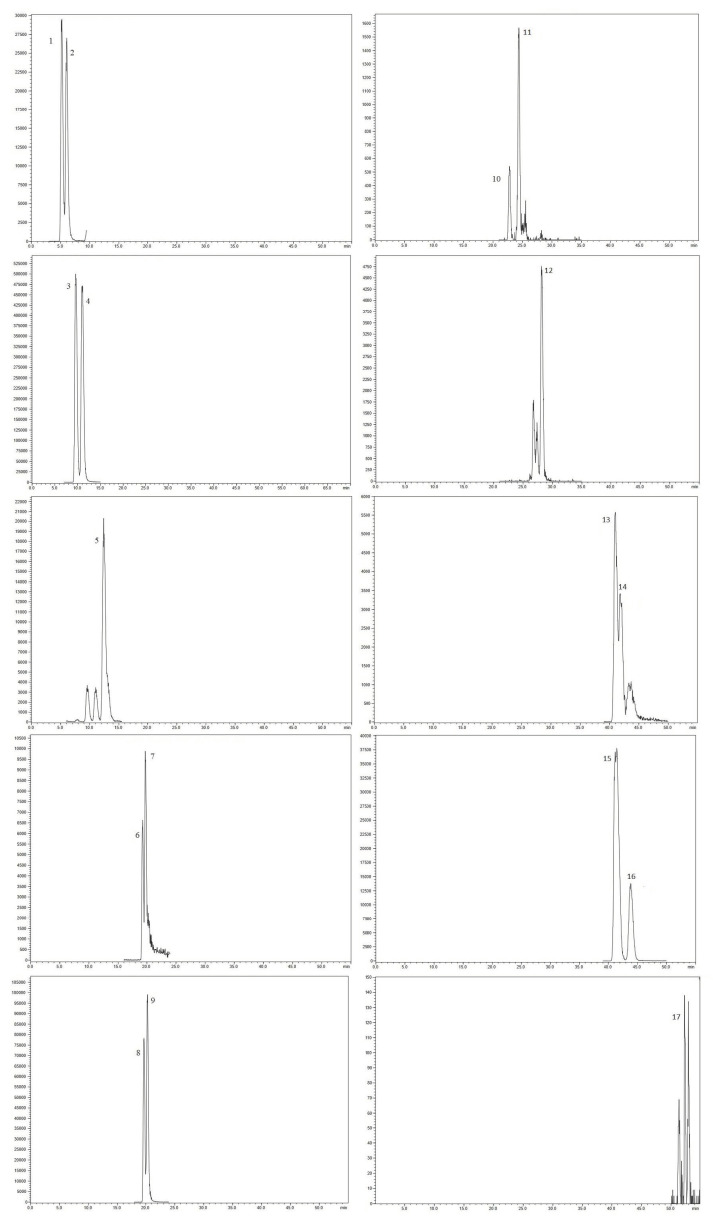
Total ion chromatograms (TIC of MRM) of pyrrolizidine alkaloids present in methanol extract of comfrey (*Symphytum officinale*) root obtained from a herbal store (HR2). Compounds were separated out and tentatively identified via HPLC-MS/MS as presented in Table 1.

Chromatographic data of all pyrrolizidine alkaloids (t_R_, [M + H]^+^ (*m*/*z*), MS^2^ (*m*/*z*)) identified in comfrey roots and leaves are presented in Table 1. The reported elution order in Table 1 can be viewed on Figure 2, and it is mostly consistent with that reported by Trifan et al. [8].

Identification of sarracinyl derivatives, namely 7′-sarracinyl-9-trachelantylretronecine *N*-oxide (**10**) and 7′-sarracinyl-9-viridifloryloretronecine *N*-oxide (**11**) and their differentiation from echimidine *N*-oxide (**12**), was based on data from the literature on PAs from comfrey root [8] and two studies on the presence of PAs in herbal teas and honey [22,27]. On this basis, the characteristic transitions 414 > 254, 414 > 352, and 414 > 396 (m/z) for echimidine *N*-oxide (**12**) and 414 > 270 (m/z) for sarracinyl derivatives (**10**, **11**) were determined. In sample NR1, fragmentation ions at m/z 270 and m/z 396 were observed to have similar retention times, confirming that both compounds elute together.

As a result of using a Kinetex C-18 column instead of a Gemini C-18 column, an elution order of the symphytine/symlandine derivatives was observed that differed from that previously reported by Trifan et al. [8]. The observed elution order of symphytine/symlandine derivatives was consistent with that previously described for intermedine/lycopsamine derivatives, namely free alkaloid > *N*-oxide > acetyl derivative [25]. Symphytine and symlandine, and their *N*-oxides separated out in order of increasing retention times: symphytine (**13**) > symlandine (**14**) > symphytine *N*-oxide (**15**) > symlandine *N*-oxide (**16**).

Compound **17**, which yielded 4 peaks in MRM mode (except root sample HR3, in which **17** had 3 peaks) was tentatively identified as 3’-acetylsymphytine *N*-oxide (**17**) and/or its isomer, i.e., 3’-acetylsymlandine *N*-oxide [8]. Due to their very low intensity, caused by a very low concentration in the analyzed plant material, it was not possible to distinguish individual isomers.

### 2.3. Semi-Quantitative Analysis of Pyrrolizidine Alkaloids in Comfrey Root and Leaf

Most of the identified pyrrolizidine alkaloids were present in all tested plant matrices—various samples of roots and leaves of comfrey (Table 2). The only exception was 3’-acetylsymphytine *N*-oxide (**17**) and its isomers—the compound was not detected in the two samples of comfrey roots from botanical gardens (GR1, GR2), nor in any of the analyzed comfrey leaves.

Semi-quantitative analysis of all detected pyrrolizidine alkaloids was performed based on a comparison of the peak areas of the first transition ions (quantifier ions) obtained in MRM positive ion mode. The data presented in Table 2 compare the proportions of individual pyrrolizidine alkaloids only in a single analyzed plant material and not between all analyzed samples. The reason for such presentation of the results was to show that depending on the origin associated, among others, with the growth conditions of the parent plant, comfrey roots and leaves may differ in composition of pyrrolizidine alkaloid complexes, in which various individual alkaloid compounds may predominate.

An example base peak chromatogram in MRM mode of the full alkaloid profile (root sample HR2) is available for download in Supplementary Materials—Figure S1.

The dominant compounds in all analyzed comfrey roots were intermedine (**1**), lycopsamine (**2**), and their *N*-oxides (**3**, **4**) as well as 7′-acetylintermedine (**6**) and 7′-acetyllycopsamine *N*-oxide (**7**). On the other hand, comfrey leaves contained intermedine (**1**), lycopsamine (**2**), and their *N*-oxides (**3**, **4**).

To sum up, in most of the tested roots, the dominant compounds were lycopsamine derivatives, which, according to Wang et al. [12], are more toxic than intermedine. Intermedine (**1**) was detected in higher concentrations than lycopsamine (**2**) in only two comfrey root samples, HR2 and HR3. 

Intermedine (**1**), lycopsamine (**2**), and their *N*-oxides (**3** and **4,** respectively), constitute the majority of pyrrolizidine alkaloid complexes in comfrey leaves, while other compounds occur in practically trace concentrations. Among the examined leaves, only in HL4 leaves a slightly higher content of echmidine *N*-oxide (**12**) and a slightly lower content of intermedine and lycopsamine were noted.

### 2.4. Quantitative Analysis of Pyrrolizidine Alkaloids in Comfrey Root and Leaf

In addition to the semi-quantitative analysis describing the content of individual alkaloids in the analyzed plant materials, a quantitative analysis of the dominant PAs—intermedine (**1**), lycopsamine (**2**), intermedine *N*-oxide (**3**) and lycopsamine *N*-oxide (**4**) was carried out, and their content was determined in extracts and expressed in ng/μL (corresponding to μg/mL) and presented in Table 3; and in plant raw materials (mg/g d.w.) and are presented in Table 4. The validation data are available in Supplementary Materials—Table S1.

In the examined comfrey roots originating from different sources, even the content of intermedine (**1**) differed by about 25 times (0.0077 mg/g d.w. in GR1 vs. 0.196 mg/g d.w. in the HR2 plant sample), and the content of intermedine *N*-oxide (**3**) by more than 10 times (0.0077 mg/g d.w. in GR1 vs. 13 mg/g d.w. in GR1 vs. 1.56 mg/g d.w. in HR1) (Table 4). It is worth noting that the highest content of pyrrolizidine alkaloids was found in two plant raw materials from commercial sources (herbal stores) [HR2 roots—intermedine 0.196 mg/g d.w. and lycopsamine 0.17 mg/g d.w.; HR1 roots—intermedine *N*-oxide 1.56 mg/g d.w. and lycopsamine *N*-oxide 1.61 mg/g d.w. (Table 4)]. These plant raw materials are available on the herbal product market and used without adequate information about their potential toxicity and the resulting health risks. The determined content of the four main pyrrolizidine alkaloids in comfrey roots from various Polish sources is consistent with the data from the literature [8], especially in the case of intermedine *N*-oxide (**3**) (determined content 0.131.56 mg/g d.w. vs. 0.2–1.69 mg/g d.w. reported by *Trifan* et al. [8]) and lycopsamine *N*-oxide (**4**) (determined 0.37–1.61 mg/g d.w. vs. 0.24–1.87 mg/g d.w. reported by Trifan et al. [8]) (Table 4).

In turn, two samples of comfrey roots obtained from commercial sources (HR2 and HR3) were characterized by the highest concentrations of intermedine and lycopsamine (0.196 and 0.135 mg/g d.w., respectively) (Table 4), which were also higher than those previously reported in the literature (0.1 and 0.118 mg/g d.w, respectively) [8].

As demonstrated in qualitative and quantitative studies, the analyzed comfrey leaf extracts are characterized by more reproducible metabolic profiles and a lower content of pyrrolizidine alkaloids compared to the roots. In the analyzed comfrey leaf extracts, the determined content of intermedine (**1**) ranged from 0.015 to 0.03 mg/g d.w., lycopsamine (**2**) from 0.026 to 0.05 mg/g d.w., intermedine *N*-oxide (**3**) from 0.026 to 0.062 mg/g d.w. and lycopsamine *N*-oxide (**4**) from 0.026 to 0.11 mg/g d.w. (Table 4).

As mentioned in previous reports [16], comfrey leaves contain significantly fewer PAs, and in our study the sum of the four dominant compounds ranged from 0.11 to 0.24 mg/g d.w. and was approximately 6 to 14 times lower than their content determined in the roots (0.60–3.28 mg/g d.w.) (Table 4).

### 2.5. Factor Analysis of Pyrrolizidine Alkaloids Distribution in the Roots and Leaves of Comfrey

**Figure 3:**
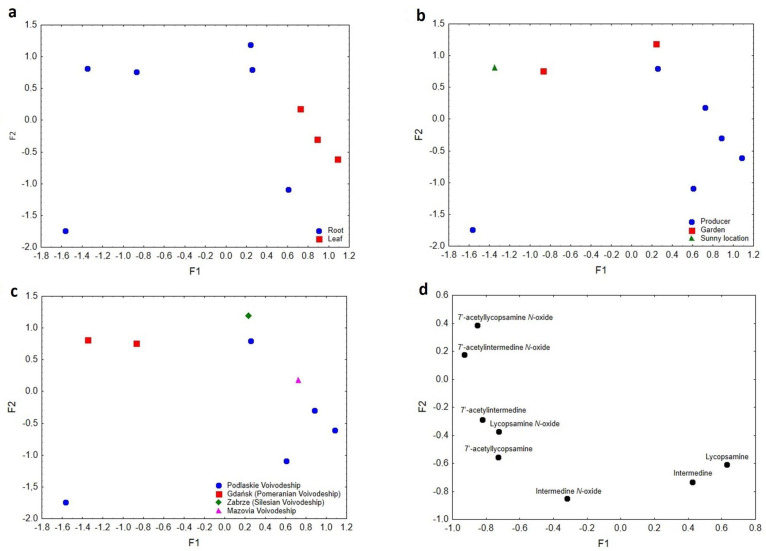
(**a**) Scatterplot of object samples of two factors regarding anatomical parts of comfrey. (**b**) Scatterplot of objects samples of two factors in view of cultivation conditions. (**c**) Scatterplot of objects samples of two factors in view of geographical origin. (**d**) Scatterplot of loadings for the determined substances in all the analyzed samples.

The factor analysis (FA) used was based on both root and leaf comfrey samples. The results of the analyses are shown in Figure 3a–d. Factor one (F1) accounted for 49.96% of the explained variance, and factor two (F2) for 29.21%. The cumulative value of the explained variance was 79.17%. The eigenvalues for F1 and F2 were 3.99 and 2.34, respectively.

As can be seen in Figure 3a–d, the comfrey samples differed in several respects:

- the anatomical part of the plant (root–leaf)
- growing conditions (differentiating between garden conditions and those used by producers)
- the geographical origin of the samples (various regions of Poland)

In Figure 3a, the comfrey samples have been differentiated according to the anatomical part of the plant (root–leaf). Factor 1 was mainly responsible for this diversification. Its high values distinguished the comfrey leaf samples, which have been described in terms of lycopsamine and intermedine. Low values of F1 characterized root samples, and 7′-acetylintermedine *N*-oxide, 7′-acetyllycopsamine *N*-oxide, 7′-acetylintermedine, 7′-acetyllycopsamine, intermedine *N*-oxide, and lycopsamine *N*-oxide were responsible for their separation (Figure 3d).

In contrast, in Figure 3b, F2 was responsible for the separation of samples due to cultivation conditions. High F2 values distinguished samples grown in gardens or a sunny location compared to low F2 values, where other cultivation conditions (less sunlight) were used by comfrey producers. 7′-acetylintermedine *N*-oxide and 7′-acetyllycopsamine *N*-oxide were responsible for the distinction between samples grown under garden or sunny conditions. This suggested a probable relationship between growing conditions (e.g., insolation) on the content of 7′-acetylintermedine *N*-oxide and 7′-acetyllycopsamine *N*-oxide in the plant root (Figure 3d). Moreover, despite the fact that the leaves are more exposed to sunlight, they show greater stability of the analyzed compounds compared to the roots, where the variability of these substances is much greater.

The resulting comfrey database was also analysed in view of geographic origin. The data presented in Figure 3c illustrated the distribution of comfrey samples within different regions of Poland. High F1 values characterized samples from Podlaskie Voivodeship, Zabrze (Silesian Voivodeship), and Mazovia Voivodeship. Low ones, on the other hand, were typical of samples originating from Gdańsk (Pomeranian Voivodeship). It was possible to observe a considerable impact of the climatic conditions prevalent in different parts of Poland on the synthesis of specific compounds in the plant when taking into consideration the locations of the different regions of the sample collection. The comfrey samples from Gdańsk (northern part of Poland, Pomeranian Voivodeship) were characterized by substances such as 7′-acetylintermedine *N*-oxide, 7′-acetyllycopsamine *N*-oxide and partially 7′-acetylintermedine (Figure 3d). Samples from the south-eastern part of Poland (Podlaskie Voivodeship, Zabrze (Silesian Voivodeship) and Mazovia Voivodeship) were attributed high F1 values and were characterized by lycopsamine *N*-oxide, 7′-acetyllycopsamine, intermedine *N*-oxide, lycopsamine and intermedine.

## 3. Discussion

Comfrey products are widely recommended by herbalists and used by patients in the self-treatment of musculoskeletal disorders. Considering the popularity of comfrey products and the widely available knowledge on the hepatotoxicity of the pyrrolizidine alkaloids contained in this plant, it seems that clinical studies on the toxicity of herbal substances derived from comfrey are quite neglected. Data on the hepatotoxicity of PAs present in comfrey refer to the results of studies in animal models [5,28,29] and reports of clinical cases after the use of certain herbal supplements containing misidentified raw plant materials [30].

It seems relatively recently, only 5 years ago, that the complex of pyrrolizidine alkaloids in comfrey root was fully recognized and further PAs were identified [8]. The first data on the penetration of alkaloids, mainly lycopsamine, through the human skin were also published [15]. Comfrey root has been used in traditional medicine for thousands of years, but in phytotherapy, the range of its applications has been significantly limited due to the toxicity of alkaloids, although data on toxicity seem incomplete and further research is needed in this area. The estimation of potential side effects in relation to the observed therapeutic benefits seems important because this herbal substance is often used in self-medication or is a component of herbal products, such as cosmetics or dietary supplements, which are not subject to strict quality requirements.

There are few studies in the literature on the PA profile of comfrey, as these compounds occur in very low concentrations and are characterized by low volatility and lack of chromophores, which complicates their analysis using techniques such as GC and HPLC with UV/Vis or DAD detectors [18,19]. The stereoisomeric nature of comfrey PAs causes problems with their separation, which results in the quantification of some PAs as pairs of isomers rather than single compounds [8]. The developed LC-MS/MS method enables the effective separation of all PAs isomers and, as a result, the quantitative determination of individual alkaloid compounds, especially those dominant in the comfrey root—intermedine, lycopsamine, and their *N*-oxides. Due to the lack of standard substances for the majority of alkaloids identified in the root and leaves of comfrey, their content was determined using a semi-quantitative method [13].

The hepatotoxicity of PAs is mainly attributed to three pathways of action: (1) excessive formation of ROS via cellular oxidative stress; (2) promoting apoptosis via the mitochondrial intrinsic pathway, partly through the formation of pyrrole-protein adducts in hepatocytes, which leads to a decrease in intracellular ATP levels and disruption of mitochondrial homeostasis; (3) inducing stagnation of bile acids in the liver, which results in the disruption of bile acid homeostasis and liver damage. The greatest toxic potential was characterized by ring-opened retronecine diesters (such as symphytine, symlandine), followed by macrocyclic diesters, then monoesters (e.g., lycopsamine, intermedine) [11,12]. The latter pose the greatest threat because they are present in the highest concentrations in the comfrey root [11,12].

The toxicity levels of the four major PAs (intermedine, lycopsamine, and their *N*-oxides) were recently compared by Wang et al. [12,13] on human (HepD, HepG2) and murine (primary hepatocytes, H22) liver cell lines. It has been shown that these compounds, even at a concentration of 20 μg/mL, can inhibit cell viability and have cytotoxic effects, with the highest toxicity to human cells being characterized by lycopsamine and its *N*-oxide [12].

The content of intermedine and lycopsamine determined in the tested plant material was almost 10 times lower than the indicated IC_50_ values—maximum 7.83 and 6.95 μg/mL vs. IC_50_ 56.61 and 65.11 μg/mL [12]. However, the concentrations of intermedine *N*-oxide in extracts from the two root samples HR1 and HR3 were higher than the reported IC_50_ values for HepD and HepG2 cells—62.30 and 60.85 μg/mL, respectively vs. IC_50_ of 56.38 and 57.32 μg/mL [12]. Similarly, slightly exceeded IC_50_ values were observed in the same samples for lycopsamine *N*-oxide—64.5 and 58.3 μg/mL vs. IC_50_ 49.11 and 46.43 μg/mL [12] (calculations to be downloaded from additional materials—Table S2).

A mixture of the two pyrrolizidine alkaloids, intermedine and lycopsamine, was revealed to have more significant HepD cell toxicity compared to the single compounds, especially at the lower concentrations tested. Cell viability after treatment with 75 μg/mL of intermedine or lycopsamine was inhibited to 47% and 48.8%, respectively, while the mixture of both compounds used at the same concentration reduced it to 32.9% [13]. While the cited studies did not include pyrrolizidine alkaloid *N*-oxides, the possibility of a similar synergy, which in combination with a high content of these compounds could significantly increase the toxicity of comfrey, cannot be ruled out. Therefore, further research is needed on the toxicity of the pyrrolizidine alkaloid complex in comfrey extracts, taking into account the potential synergistic effects of these compounds.

The EMA restricts the use of comfrey products to external use on intact skin only, setting a daily dose limit of 0.35 μg/day [5]. So far, the only studies on the penetration of PAs (mainly lycopsamine) through human skin show a low permeability, below 1% but up to 4.9% in the “worst case scenario” [14,15]. The content of PAs, as the sum of the four dominant compounds characterized by the highest toxicity [12], in the analyzed samples ranges from 11.22 to 56.92 μg/mL of the plant extract, except for two comfrey roots obtained from commercial sources (HR1 and HR3), where it reaches about 130 μg/mL. In this case, even with low skin permeability of 1% (excluding other PAs present in much lower concentrations), the daily dose may be exceeded by less than 1 mL of the extract used.

According to the EMA assessment report on *Symphytum officinale* L., radix medicinal products registered in the EU may contain 10–35% liquid extract from comfrey roots or 20% tincture in ointments [1]. Considering the cited data on the possible “worst case scenario” of lycopsamine penetration through the skin and the showed large differences in PA content in comfrey roots, products with the highest content of comfrey root may pose a health risk.

The obtained results of studies on the content of pyrrolizidine alkaloids in comfrey roots indicate that the safety of their use is not certain and emphasize the importance of their standardization. The fact that the concentration of PAs in comfrey roots, depending on the origin, can vary up to 25 times, proves that products containing comfrey extracts cannot be considered as safe. Especially, when the highest concentrations of PAs were determined in comfrey roots, commercially available and sold directly in herbal shops without additional information about the ranges of their safe use.

Another interesting result of the conducted experiments is the disclosure of a very stable profile of alkaloid compounds in comfrey leaves, independent of the origin of the plant raw material. Their content in comfrey leaves is much lower compared to the root.

The use of factor analysis confirmed the differences between PA complexes in comfrey depend on the anatomical part of the plant, its growing conditions, and geographical origin. FA can be a highly effective method for executing a more comprehensive examination of comfrey samples (both roots and leaves), as well as discovering the effects of regional characteristics and growing conditions on the PAs distribution.

Further studies of comfrey leaves are needed in terms of chemical composition and determination of its therapeutic potential, and their results should be taken into account by regulatory institutions. Currently, comfrey leaf according to EMA is not an active herbal substance, although the extract from the above-ground parts of another species of comfrey—*Symphytum × uplandicum* is used for therapeutic purposes as an ingredient in an ointment (Traumaplant^®^) containing 10% comfrey leaf extract.

## 4. Materials and Methods

### 4.1. Chemicals

Acetonitrile Lichrosolv^®^, methanol Lichrosolv^®^, and chloroform were purchased from Merck (Darmstadt, Germany). Formic acid LC-MS LichroPur 97.5–98.5%, was purchased from Sigma-Aldrich (Steinheim, Germany). Ultra-pure water for LC-MS analysis was obtained using Direct-Q^®^ Water Purification System (Merck Millipore; Darmstadt, Germany).

Intermedine, lycopsamine, and intermedine *N*-oxide standards were obtained from Planta Analytica LLC (New Milford, CT, USA).

### 4.2. Plant Material and Extraction

The plant material for the study was the roots and leaves of *Symphytum officinale* L. (Boraginaceae) obtained from various sources—from plants growing in natural habitat and cultivated in gardens and from commercial sources (herbal shops). Of the 6 comfrey roots intended for testing, 2 were collected from plants grown in botanical gardens, namely, the Municipal Botanical Garden in Zabrze, Silesian Voivodeship (Poland) (GR1), and the Medicinal Plant Garden of the Medical University of Gdańsk, Pomeranian Voivodeship (Poland) (GR2), and 1 was collected from natural habitat—Gdańsk, coordinates: 54°23′37.9″N 18°31′03.0″E, Pomeranian Voivodeship (Poland) (NR1). Of these roots, 3 (anonymized as samples HR1-HR3) were purchased from herbal shops and originated from plants grown in Podlaskie Voivodeship (Poland). All 3 comfrey leaves were purchased from herbal stores [HL2, HL3—both collected in Podlaskie Voivodeship (Poland), HL4 in Mazovia Voivodeship (Poland)]. The voucher samples were deposited at the Department of Pharmacognosy of the Medical University of Gdańsk (Gdańsk, Poland).

Dried comfrey roots (1 g) were powdered and extracted with methanol (25 mL) for 15 min under reflux at 60 °C. Extracts from dried and powdered comfrey leaves (5 g) pre-treated with chloroform were prepared by extraction in Soxhlet apparatus with methanol (50 mL).

### 4.3. Separation Conditions

The chromatographic analysis was carried out with an HPLC-MS/MS Shimadzu system (Shimadzu Corp., Kyoto, Japanb), which consists of two pumps LC-20AD, a degasser DGU-20A5, a semi-micro mixer, an autosampler SIL20AC _XR_, a column oven CTO-20AC, a controller CBM-20A, a valve unit FCV-20AH_2_, a nitrogen generator PEAK Scientific GeniusXE35 230, a mass detector LCMS-8040 (Shimadzu Corp., Kyoto, Japan).

A triple-quadrupole LCMS 8040 mass spectrometer equipped with an electrospray ionization (ESI) interface, operated in positive mode, was used for mass spectrometric analysis. The operating conditions for MS analysis were: DL temperature 250 °C, heat block temperature 400 °C, nebulizing gas flow 3 L/min, drying gas flow 15 L/min, CID gas pressure 230 kPa, detector voltage 1.9 kV, interface voltage 4.5 kV for (+) ESI. Dwell time was 100 ms. Data acquisition was performed with the software LabSolutions version 5.89 (Copyrights© 2008–2016 Shimadzu Corporation, Kyoto, Japan).

Separation of analytes was performed on a Kinetex ^®^ 2.6 µm C18 100 Å column (100 mm × 2.1 mm) equipped with a precolumn. In optimized conditions the mobile phase consisted of solvent A (0.1 mL of formic acid in 100 mL of water) and solvent B (water: acetonitrile 50:50 *v*/*v* mixture with 0.1 mL of formic acid) using the following gradient: 0 min 10% B. 1.50 min 10% B, 2 min 8% B, 3 min 8% B, 9 min 0% B, 10 min 10% B, 13.00 min 10% B, 15.00 min 18%, 23.00 min 25%, 31.00 min 25%, 33.00 min 30%, 45.00 min 30%, 46.00 min 30%, 55.00 min 70%, 65.00 min 100%, 67.00 min 100%, 68.0 min 10%, 83.01 min 10%. The flow rate was 0.2 mL/min. Temperature of analysis was set at 25 °C. Procedure was based on a modified version of Altamirano et al. [11]’s method. Injection volume was 1 µL.

### 4.4. Qualitative Analysis

Analysis of the three main PAs, namely intermedine, lycopsamine, intermedine *N*-oxide, was based on comparison of MRM transitions and retention times with used standard compounds. Other compounds: lycopsamine *N*-oxide, dihydrointermedine *N*-oxide, dihydrolicopsamine *N*-oxide (or stereosisomers), 7-acetylintermedine, 7-acetyllycopsamine, 7-acetylintermedine oxide, 7-acetylintermedine *N*-oxide, 7′-sarracinyl-9-trachelanthylretronecine *N*-oxide (or stereoisomers), 7′-sarracinyl-9-viridiflorylretronecine *N*-oxide (or stereoisomers), echimidine *N*-oxide, symphytine, symlandine, symphytine *N*-oxide, symlandine *N*-oxide, 3′-acetylsymphytine *N*-oxide, 3′-acetylsymlandine *N*-oxide, were identified based on transitions in MRM mode described in literature [8]. The collision energy (CE) for the most intense transitions was estimated by checking the intensity of transitions in the energy range of 15–45 eV in steps of 5 eV. For each transition, the energy with which the transition is most intense was selected.

### 4.5. Quantitative and Semi-Quantitative Analysis

Quantitative analysis of intermedine, lycopsamine, and intermedine *N*-oxide in investigated samples was conducted by standard addition method in MRM positive ion mode. Reference compounds solutions were prepared by dissolving 1 mg of intermedine, lycopsamine or lycopsamine *N*-oxide in 1 mL of methanol. Next, the stock solutions containing a mixture of three standard compounds, appropriately diluted in methanol, were prepared in order to plot the calibration curves in a range of 1–9.25 μg/mL for intermedine, 2–20 μg/mL for lycopsamine: and 1–16 μg/mL for intermedine *N*-oxide: to be further added to diluted analyzed plant extracts. The used dilutions of analyzed samples (up to 1:30, depending on type of comfrey sample) and the range of calibration curve were selected based on the expected content of quantified PAs. Due to the lack of reference compound, quantification of lycopsamine *N*-oxide was based on the calibration curve of intermedine *N*-oxide.

The HPLC-MS/MS method developed for the purposes of quantitative analysis was validated by determining calibration curves, linear regression, limits of quantitation (LOQ), limits of detection (LOD), repeatability, and recovery of the analyzed compounds, which were estimated according to ICH guidelines [31].

The semi-quantitative analysis of all detected pyrrolizidine alkaloids was conducted in MRM positive ion mode, based on the comparison of peak areas of the first MRM transition (MS^2^).

### 4.6. Statistical Analysis

The mean differences between pyrrolizidine alkaloids concentrations were established via one-way analysis of variance (ANOVA), followed by Tukey’s multiple comparison tests. Differences were considered significant at *p* < 0.05. All statistical analyses were performed using Statistica 12 software (StatSoft, Krakow, Poland).

Factor analysis (FA) was carried out based on data obtained from semi-quantitative analysis of PAs in the extracts from roots and leaves of comfrey and performed in MRM mode. The database obtained was developed using Statistica 13.3 (TIBCO Software Inc., Palo Alto, CA, USA).

## 5. Conclusions

So far, pyrrolizidine alkaloids have been analyzed for hepatotoxicity mainly as single compounds, without taking into account the synergistic effect of other compounds contained in comfrey extracts, e.g., rosemary acid, with hepatoprotective properties. Due to the demonstrated large variation in the composition of PAs in comfrey roots depending on the origin, more attention should be paid to the quality assessment and standardization of comfrey products in the context of their safety. Moreover, additional research needs to be carried out on comfrey leaves—their chemical profile and therapeutic value—to assess their potential as a possible alternative to comfrey roots.

Taking into account the results of research from recent years [2,10,15], it seems that the restrictive EMA regulations limiting the use of comfrey root in particular should be revised. At the same time, further studies of comfrey leaves and roots are needed to fully identify and quantify the profile of pyrrolizidine alkaloids and the toxic potential of individual compounds against liver cells. In addition, more detailed studies are needed regarding the permeability of these compounds through the skin and evaluation of their toxicity when applied directly to the skin, i.e., to human skin cells.

## Figures and Tables

**Table 1:** Chromatographic data of pyrrolizidine alkaloids (t_R_ min, [M + H]^+^ (*m*/*z*), MS^2^ (*m*/*z*)) present in methanol extracts from roots and leaves of comfrey (*Symphytum officinale*). Identification of compounds was based on data from the literature [8,21,24,25] and comparison with reference substances (*).

No.	Compound Name	Retention Time (min)	Precursor Ion [M + H]^+^ (*m*/*z*)	MS^2^ (*m*/*z*) (CE [eV])
1	Intermedine *	5.25	300.1	94.1 (−28), 138.05 (−19). 156.1 (−30)
2	Lycopsamine *	6.07	300.1	94.1 (−28), 156.05 (−30), 138.1 (−20)
3	Intermedine *N*-oxide *	9.62	316.1	172.05 (−28), 138.05 (−29), 111 (−42)
4	Lycopsamine *N*-oxide	11.04	316.1	172.05 (−28), 138.05 (−29), 111 (−42)
5	Dihydrointermedine *N*-oxide/ Dihydrolycopsamine *N*-oxide	12.48/13.16	318.2	174.1 (−30), 113.05 (−40), 156.1 (−40)
6	7′-acetylintermedine	19.28	342.2	120.07 (−25), 180.09 (−15), 198.07 (−30)
7	7′-acetyllycopsamine	19.66	342.2	120.08 (−25), 180.1 (−15), 198.08 (−30)
8	7′-acetylintermedine *N*-oxide	19.76	358.1	214.01 (−30), 180.09 (−30), 137.08 (−30)
9	7′-acetyllycopsamine *N*-oxide	20.25	358.1	214.01 (−30), 180.1 (−30), 137.07 (−30)
10	7′-sarracinyl-9- trachelanthylretronecine *N*-oxide	22.79	414.2	270.1 (−30), 120.07 (−40), 137.08 (−40)
11	7′-sarracinyl-9- viridiflorylretronecine *N*-oxide	24.31	414.2	270.1 (−30), 137.08 (−35), 120.08 (−45)
12	Echimidine *N*-oxide	26.20/27.27/28.14	414.2	254.1 (−30), 396.07 (−25), 352.1 (−25), 137.08 (−30)
13	Symphytine	40.92	382.2	120.08 (−25), 238.1 (−30), 220.1 (−15), 138.08 (−35)
14	Symlandine	41.86	382.2	120.07 (−25), 220.1 (−15), 238.1 (−30), 138.08 (−35)
15	Symphytine *N*-oxide	41.14	398.2	254.1 (−30), 137.07 (−35), 220.1 (−15)
16	Symlandine *N*-oxide	43.83	398.2	254.1 (−30), 137.08 (−35), 220.1 (−15)
17	3′-acetylsymphytine *N*-oxide and its isomers	51.28/52.79/52.31/52.96	440.2	254.1 (−30), 220.1 (−30), 137.07 (−40), 120.07 (−35)

**Table 2:** Distribution of pyrrolizidine alkaloids in comfrey roots and leaves based on semi-quantitative analysis performed in MRM mode. The proportions between compounds are related to their first fragmentation ion peak areas in the plant sample—the table presents differences in PA content in the analyzed plant material (it does not characterize differences between analyzed plant materials obtained from various sources).

No.	Compound Name	Roots						Leaves		
		GR1	GR2	HR1	HR2	HR3	NR1	HL2	HL3	HL4
1	Intermedine	+	++	+++	+++	++	++	++++	+++	++
2	Lycopsamine	++	++	+++	+++	+++	++	++++	++++	+++
3	Intermedine *N*-oxide	+++	++++	++++	++++	++++	+++	++++	+++	+++
4	Lycopsamine *N*-oxide	++++	++++	++++	++++	++++	++++	++++	+++	++++
5	Dihydrointermedine *N*-oxide/ Dihydrolycopsamine *N*-oxide	+	+	+	+	+	+	+	+	+
6	7′-acetylintermedine	+	+	++	++	++	++	+	+	+
7	7′-acetyllycopsamine	++	+	++	++	++	++	+	+	+
8	7′-acetylintermedine *N*-oxide	++	+++	++	+++	+++	+++	+	+	+
9	7′-acetyllycopsamine *N*-oxide	+++	+++	++	+++	+++	++++	+	+	+
10	7′-sarracinyl-9- trachelanthylretronecine *N*-oxide	+	+	+	+	+	+	+	+	+
11	7′-sarracinyl-9- 7′-sarracinyl-9- viridiflorylretronecine *N*-oxide	+	+	+	+	+	+	+	+	+
12	Echimidine *N*-oxide	+	+	+	+	+	+	+	+	++
13	Symphytine	+	+	++	+	+	+	+	+	+
14	Symlandine	+	+	++	+	+	+	+	+	+
15	Symphytine *N*-oxide	+	+	++	+	+	+	+	+	+
16	Symlandine *N*-oxide	+	+	++	+	+	+	+	+	+
17	3′-acetylsymphytine *N*-oxide and its isomers	–	–	+	+	+	+	–	–	–

++++—dominant compound, +++—major compound, ++—minor compound, +—trace compound, –—compound not detected.

**Table 3:** Content (ng/mL) of pyrrolizidine alkaloids present in methanol extracts from roots and leaves of comfrey (*Symphytum officinale*) based on the standard addition method and determined by HPLC-MS/MS method in MRM mode.

Plant Material	Intermedine [ng/mL]	Lycopsamine [ng/mL]	Intermedine *N*-oxide [ng/mL]	Lycopsamine *N*-oxide [ng/mL]
GR1	0.307 ± 0.034	2.24 ± 0.17	5.15 ± 0.30	20.42 ± 1.23
GR2	0.832 ± 0.085	1.54 ± 0.16	8.09 ± 0.59	13.49 ± 0.4
HR1	2.07 ± 0.21	2.51 ± 0.12	62.30 ± 8.76	64.5 ± 2.5
HR2	7.83 ± 0.81	6.95 ± 0.43	8.10 ± 0.81	14.65 ± 0.83
HR3	5.38 ± 0.52	6.31 ± 0.23	60.85 ± 2.70	58.3 ± 7.9
NR1	1.56 ± 0.18	2.82 ± 0.28	18.80 ± 1.10	33.74 ± 1.36
HL2	1.49 ± 0.16	2.59 ± 0.30	2.63 ± 0.15	4.500 ± 0.066
HL3	3.00 ± 0.10	3.15 ± 0.19	2.63 ± 0.06	2.61 ± 0.11
HL4	2.15 ± 0.19	4.99 ± 0.44	6.22 ± 0.12	10.9 ± 0.360

**Table 4:** Content (mg/g d.w.) of pyrrolizidine alkaloids present in comfrey roots and leaves based on the standard addition method and determined by HPLC-MS/MS method in MRM mode.

Plant Material Origin	Intermedine [mg/g d.w.]	Lycopsamine [mg/g d.w.]	Intermedine *N*-oxide [mg/g d.w.]	Lycopsamine *N*-oxide [mg/g d.w.]	Σ_Pas_ *
GR1 ^a^	0.0077 ± 0.00090 ^bcdefghi^	0.0560 ± 0.0042 ^bdefgh^	0.1287 ± 0.0075 ^bcdefghi^	0.5105 ± 0.0307 ^bcdefghi^	0.70 ± 0.043
GR2 ^b^	0.0208 ± 0.0021 ^acdefh^	0.0385 ± 0.0040 ^acdefgi^	0.2022 ± 0.0147 ^bcefghi^	0.3372 ± 0.0100 ^acefghi^	0.60 ± 0.31
HR1 ^c^	0.0517 ± 0.0052 ^abdefghi^	0.0627 ± 0.0030 ^bdefghi^	1.5575 ± 0.1150 ^abdfghi^	1.6125 ± 0.0625 ^abdefghi^	3.28 ± 0.19
HR2 ^d^	0.1957 ± 0.0202 ^abcefghi^	0.1737 ± 0.0107 ^abcefghi^	0.2057 ± 0.0147 ^acefghi^	0.3662 ± 0.0207 ^acefghi^	0.94 ± 0.0665
HR3 ^e^	0.1345 ± 0.0130 ^abcdfghi^	0.1577 ± 0.0057 ^abcdfghi^	1.5212 ± 0.0675 ^abdfghi^	1.4575 ± 0.1975 ^abcdfghi^	3.27 ± 0.284
NR1 ^f^	0.0390 ± 0.0045 ^abcdeghi^	0.0705 ± 0.0070 ^abcdeghi^	0.4700 ± 0.0275 ^abcdeghi^	0.8435 ± 0.0340 ^abcdeghi^	1.42 ± 0.073
HL2 ^g^	0.0149 ± 0.0016 ^acdefh^	0.0259 ± 0.003 ^abcdefi^	0.0264 ± 0.0017 ^abcdef^	0.0449 ± 0.0007 ^abcdef^	0.11 ± 0.0070
HL3 ^h^	0.0300 ± 0.0010 ^abcdefgi^	0.0315 ± 0.0019 ^acdefi^	0.0263 ± 0.0006 ^abcdef^	0.0261 ± 0.0011 ^abcdefi^	0.11 ± 0.0046
HL4 ^i^	0.0215 ± 0.0019 ^acdefh^	0.0499 ± 0.0044 ^bcdefgh^	0.0622 ± 0.0012 ^abcdef^	0.1090 ± 0.0036 ^abcdefh^	0.24 ± 0.11

* Sum of quantified pyrrolizidine alkaloids mean ± standard deviation; ^abcdefghi^ Statistically significant differences in pyrrolizidine alkaloid contents (*p* < 0.05) between analyzed plant materials; differences are indicated by the same letter (Tukey’s test).

## Data Availability

Not applicable.

## Supplementary Materials

The following supporting information can be downloaded at: https://www.mdpi.com/article/10.3390/molecules28166171/s1, Figure S1: HPLC-MS/MS base peak chromatogram (MRM mode) of pyrrolizidine alkaloids present in methanol extract of comfrey (*Symphytum officinale*) root obtained from a herbal store (HR2)—the numbering of the peaks corresponds to numbers of PAs given in Table 1. Table S1: Validation data for quantitative analysis of pyrrolizidine alkaloids by proposed HPLC-MS/MS method; Table S2: Mean IC_50_ values of intermedine, lycopsamine, intermedine *N*-oxide and lycopsamine *N*-oxide in HepD and HepG2 cells reported by Wang et al. [12] calculated to μg/mL.

## Sample Availability

Samples of the analyzed plant materials are available from the authors and beforementioned sources. Information on the anonymized herbal companies can be given on the request.
