# Supplementary material for: LC-MS/MS Evaluation of Pyrrolizidine Alkaloids Profile in Relation to Safety of Comfrey Roots and Leaves from Polish Sources
Source: Molecules. 2023 Aug 21;28(16):6171. doi: 10.3390/molecules28166171 (PMC10459857; doi:10.3390/molecules28166171)
Supplement: Supplementary file 1 [file molecules-28-06171-s001.zip › molecules-2502691-supplementary.pdf]

LC-MS/MS Evaluation of Pyrrolizidine Alkaloids Profile in Relation to Safety of Comfrey Roots and Leaves from Polish Sources

Katarzyna Kimel [1], Sylwia Godlewska [1], Michał Gleńsk [2], Katarzyna Gobis [3], Justyna Aśko [4], Małgorzata Grembecka [4] and Mirosława Krauze-Baranowska [1,*]

[1] Department of Pharmacognosy with Medicinal Plants Garden, Faculty of Pharmacy, Medical University of Gdańsk, 107 Hallera St., 80-416 Gdansk, Poland; katarzyna.kimel@gumed.edu.pl (K.K.); sylwia.godlewska@gumed.edu.pl (S.G.)
[2] Department of Pharmacognosy and Herbal Medicines, Wroclaw Medical University, 211A Borowska St., 50-556 Wrocław, Poland; michal.glensk@umw.edu.pl
[3] Department of Organic Chemistry, Faculty of Pharmacy, Medical University of Gdańsk, 107 Hallera St., 80-416 Gdansk, Poland; katarzyna.gobis@gumed.edu.pl
[4] Department of Bromatology, Faculty of Pharmacy, Medical University of Gdańsk, 107 Hallera St., 80-416 Gdansk, Poland
* Correspondence: miroslawa.krauze-baranowska@gumed.edu.pl

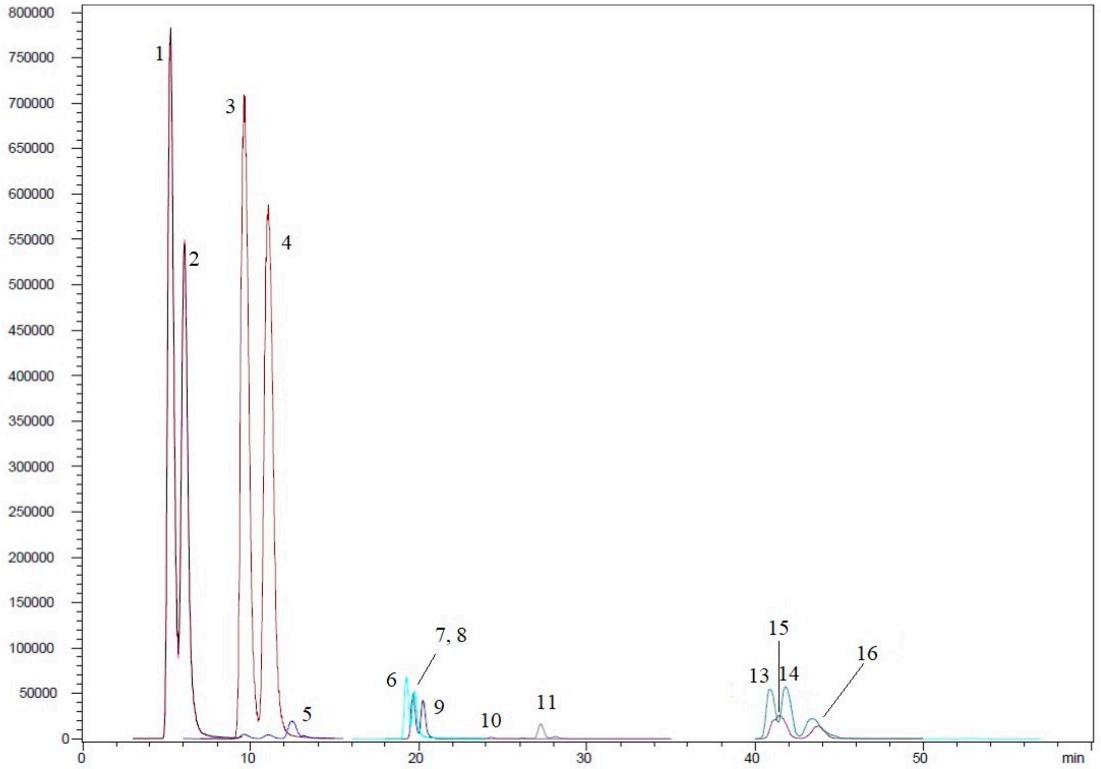

Figure S1: HPLC-MS/MS base peak chromatogram (MRM mode) of pyrrolizidine alkaloids present in methanol extract of comfrey (Symphytum officinale) root obtained from the herbal store (HR2) – the numbering of the peaks corresponds to numbers of PAs given in Table 1.

Table S1: Validation data for quantitative analysis of pyrrolizidine alkaloids by proposed HPLC-MS/MS method

Validation parameter		Intermedine	Lycopsamine	Intermedine *N*-oxide	Lycopsamine *N*-oxide
Correlation coefficients	Roots	0.9818-0.9923	0.9824-0.9984	0.9973-0.9997	-*
	Leaves	0.9810-0.9905	0.9909-0.9944	0.9901-0.9983	-*
LOD [µg/mL]	Roots	0.0026-0.0045	0.0028-0.0054	0.0027-0.0048	0.0026-0.0063
	Leaves	0.010-0.013	0.0028-0.0048	0.0032-0.0061	0.0026-0.0031
LOQ [µg/mL]	Roots	0.0086-0.015	0.0094-0.018	0.0089-0.016	0.0086-0.021
	Leaves	0.0030-0.0038	0.0094-0.016	0.0103-0.0204	0.0087-0.010
Recovery ± CV [%][1]	50%	105 ± 12	111 ± 3	112 ± 11	-
	100%	117 ± 22	112 ± 8	114 ± 12	-
Precision [CV%][2]	Intra-day	0.63	0.67	0.97	-
	Inter-day	1.18	0.98	1.00	-

*Results based on calibration curve for intermedine *N*-oxide; [1]Measured for comfrey leaf sample (HL2) (n-2); [2]Measured for comfrey leaf sample (HL2) (intra-day: n-6, interday: n-3)

Table S2: Mean IC_{50} values of intermedine, lycopsamine, intermedine *N*-oxide and lycopsamine *N*-oxide in HepD and HepG2 cells reported by *Wang* et al [12] calculated to µg/mL.

	HepD	HepG2
**Intermedine**	71.66	56.61
**Lycopsamine**	81.36	65.11
**Intermedine *N*-oxide**	49.11	46.43
**Lycopsamine *N*-oxide**	56.38	57.32
